# Role of surgery in treating epstein‐barr virus‐associated smooth muscle tumor (EBV‐SMT) with central nervous system invasion: A systemic review from 1997 to 2019

**DOI:** 10.1002/cam4.3770

**Published:** 2021-02-11

**Authors:** Ka‐Wei Lau, Yu‐Wei Hsu, Yin‐Ting Lin, Ko‐Ting Chen

**Affiliations:** ^1^ College of Medicine Chang Gung University Taoyuan Taiwan; ^2^ Department of Neurosurgery Chang Gung Memorial Hospital at Linkou Taoyuan Taiwan; ^3^ Ph D. Program in Biomedical Engineering Chang Gung University Taoyuan Taiwan

**Keywords:** CNS invasion, EBV‐SMT, immune compromised, surgical resection

## Abstract

Epstein‐Barr virus‐associated smooth muscle tumor (EBV‐SMT) is a rare mesenchymal tumor occurred almost exclusively in immunocompromised hosts. This article provides a systematic review of literature under PRISMA guideline on clinical features, treatment modalities, roles of surgical intervention, and outcomes of all 65 reported EBV‐SMTs with central nervous system (CNS) invasion. Over 95% of reported cases were immunocompromised, while human immunodeficiency virus infection and post‐organ transplantation were the most commonly associated underlying causes (near 90%). Despite a heterogeneous follow‐up period, a 1‐year survival rate of 76.0% and 5‐year survival rate of 59.6% may support the indolent and non‐deadly nature of EBV‐SMT even with CNS invasion. Immune survey and reconstruction should be conducted for every patient with CNS EBV‐SMT. Surgical resection is mostly adopted as primary treatment to obtain diagnosis and relieve compressive effect. A total resection of tumor may be beneficial if tumor was symptomatic and had intracranial invasion.

## Graphical abstract

This study systemically reviews and analyzes all reported EBV‐SMT with CNS invasion in the literature. We provide epidemiologic, therapeutic and prognostic data for patients with CNS EBV‐SMT and conclude that a higher extent of resection may provide survival benefits for treating patients with CNS EBV‐SMT, especially when tumor is single.

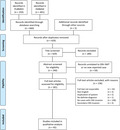

**Abbreviations**
AIDS: acquired immunodeficiency syndrome
ANED: alive with no evidence of disease
CR: complete response
EBER: Epstein‐Barr virus‐encoded small RNA
EBV: Epstein‐Barr virus
EBV‐SMT: Epstein‐Barr virus‐associated smooth muscle tumor
EOR: extent of resection
HIV: human immunodeficiency virus
PD: progressive disease
PID: primary immunodeficiency
POT: post‐organ transplantation
PR: partial response

**Importance of the study**

EBV‐SMT, a rare mesenchymal tumor, is pathologically associated with EBV infection and resulted from a transformation of normal smooth muscle cells into malignant cells. The term unknown malignant potential was once used interchangeably to describe EBV‐SMT and the less invasive clinical behavior than leiomyosarcoma indicates an indolent growth pattern of EBV‐SMT. Nevertheless, an EBV‐SMT with CNS invasion would result in neurological deficits which may jeopardize patients’ survival. This study systematically reviews and analyzes all reported EBV‐SMT with CNS invasion in the literature and also provides epidemiologic, therapeutic, and prognostic data for patients with CNS EBV‐SMT and concludes that a higher extent of resection may provide survival benefits for treating patients with CNS EBV‐SMT.

## 1 INTRODUCTION

Epstein‐Barr virus‐associated smooth muscle tumor (EBV‐SMT) is a rare subset of spindle cell tumor. The presence of EBV DNA in smooth muscle tumor was first documented in 1994, and the term EBV‐SMT was first described in 1995.^1^, ^2^, ^3^, ^4^ In the past few decades, patients at any age who were diagnosed with EBV‐SMT, have been proved to have a strong correlation with immunodeficiency.^5^, ^6^, ^7^, ^8^ Cases of EBV‐SMTs are most commonly correlated to secondary (or acquired) immunodeficiency, and the cases mainly consisted of patients with human immunodeficiency virus (HIV) infection^5^, ^9^, ^10^, ^11^, ^12^ or with post‐organ transplantation (POT) under immunosuppressants,^10^, ^11^, ^13^ while a small fraction is correlated to primary (or congenital) immunodeficiency (PID) with gene mutation, such as GATA2 and CARMIL2.^14^, ^15^ The pathogenesis of EBV‐SMTs still remains controversial, but is generally considered to be associated with an uncontrolled proliferation of EBV‐infected lymphocytes which invade and induce neoplastic transformation of smooth muscle cells.^3^, ^12^, ^16^, ^17^

Pathologically, EBV‐SMTs are mostly composed of well‐differentiated spindle cells. Meanwhile, the biphasic feature of well‐differentiated and monomorphic spindle cells and primitive‐looking oval to round cells in hypercellularity with atypia was commonly reported as a representative feature of EBV‐SMTs.^18^ Clinically, patients with EBV‐SMTs present with variable aggressiveness and clinical outcomes; unlike patients with somatic smooth muscle tumors, the behavior of EBV‐SMTs is not positively correlated their pathologic findings.^11^ Low propensity for metastasis and less aggressive outcome were noted comparing to those with leiomyosarcoma,^5^, ^7^, ^19^ while a disseminated tumor invasion was documented occasionally on patients with EBV‐SMT presenting benign pathologic features.^14^ Based on the unique pathological feature, pathophysiologic background, and uncertain malignant potential, EBV‐SMT could hardly be categorized in the family of leiomyoma or leiomyosarcoma, and a distinct entity for EBV‐SMT has been widely accepted.^18^, ^20^, ^21^

EBV‐SMTs have been documented to invade a wide variety of organs and systems, such as lung, liver, genitourinary organs, and central nervous system (CNS).^22^, ^23^, ^24^ Concurrent or consecutive multiple involvements have also been reported.^25^, ^26^, ^27^, ^28^ Despite various reports being published,^21^, ^29^, ^30^, ^31^ currently, there were no epidemiological, demographical, and survival data regarding patients with EBV‐SMT invading CNS in the literature. In the meanwhile, treatment strategies varied largely according to the underlying disease status.^11^, ^17^ For tumors with CNS invasion, oftentimes focal neurological deficits may develop and prompt surgical intervention may be needed to decompress the CNS and to obtain tissue diagnosis.^17^ Nevertheless, the impact of surgical resection on the survival on patients having EBV‐SMT with CNS invasion is largely unknown.

In this systematic review, we tried to search all of the published literatures for the studies that consisted of patients with EBV‐SMT invading CNS. The focus of this systematic review is to: (1) summarize epidemiology, demography, treatment modalities, and prognosis with regards to EBV‐SMT presented with CNS invasion, and (2) to answer to the question whether surgical resection plays a role in treating patients with EBV‐SMT invading CNS.

## 2 METHODS

As a template for the methodology, we utilized Preferred Reporting Items for and Meta‐Analyses (PRISMA) guidelines for systematic reviews. This review was not registered as a systematic review protocol in the Cochrane database.

### 2.1 Search strategy

To understand the epidemiology, disease course and prognosis of patients with EBV‐SMTs occurred in the CNS, we performed a thorough search for all publications associated with EBV‐SMT.

**FIGURE 1:**
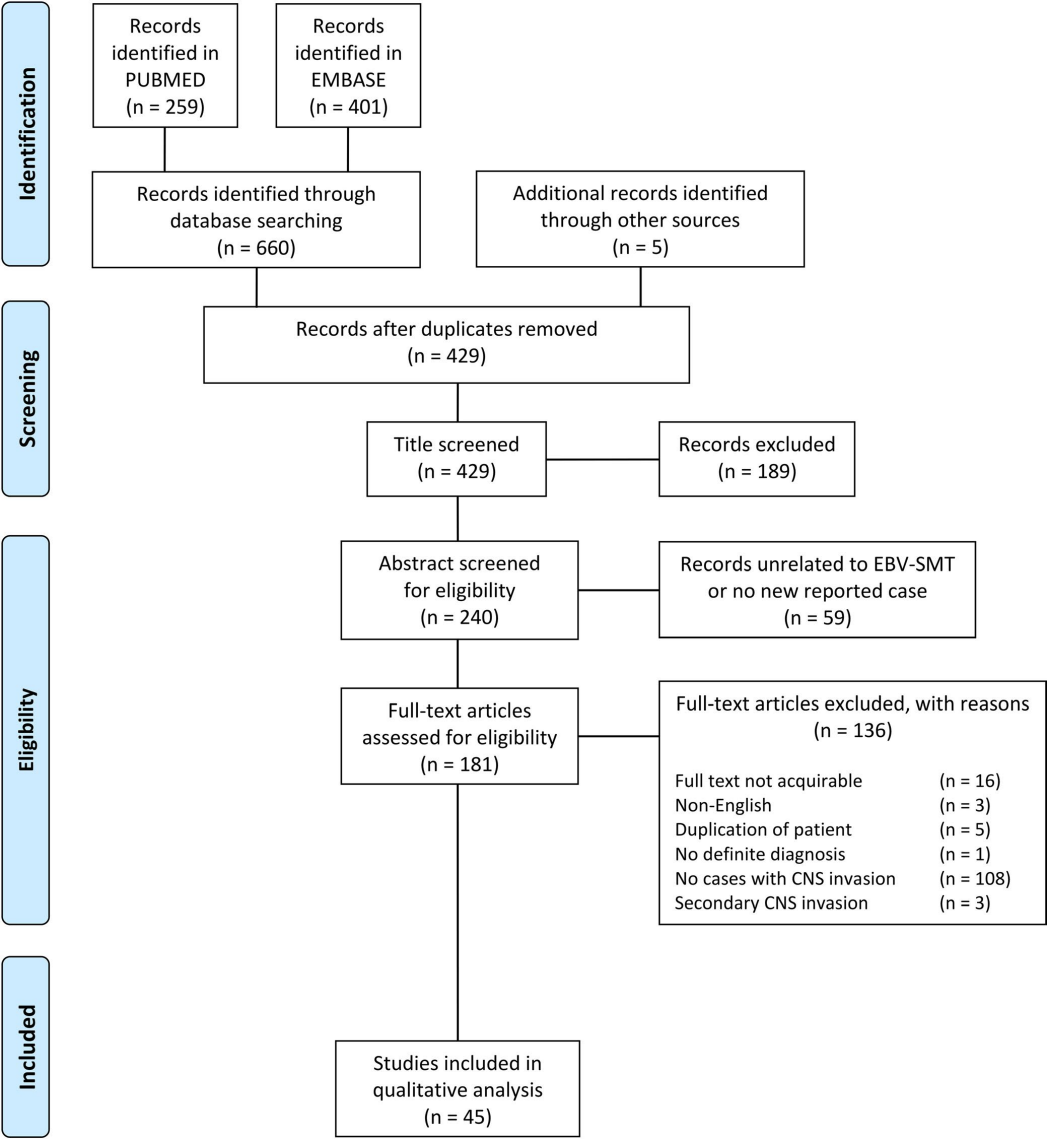
PRISMA 2009 Flow diagram. A thorough search through PUBMED and EMBASE for articles associated with EBV‐SMT was conducted, and publications in reference lists were also included as article sources. A total of 429 records were enrolled for screening, and 181 records proceeded to full‐text review. Among all, 45 records were considered suitable for qualitative analysis, while no record was found suitable for quantitative analysis

Our search was completed in two databases: PUBMED and EMBASE. We aimed to initially collect all patients reported under the diagnosis of EBV‐SMT, and further selection of patients with CNS invasion would be done by full‐text screening. Due to the uncertain malignant potential and various nomenclatures of EBV‐SMT, any leiomyoma or leiomyosarcoma associated with Epstein‐Barr virus was included in our search. The query parameter was set to [*Epstein*‐*Barr virus*‐*associated smooth muscle tumor OR (Epstein*‐*Barr virus AND (smooth muscle tumor OR leiomyoma OR leiomyosarcoma))*] in both databases. Additional studies were included according to the references of collected publications and an unpublished case of our own. According to the first documentation of EBV‐SMT in 1994, only subsequent publications were included. Our search period ended on February 17, 2019. (Figure 1).

### 2.2 Inclusion and exclusion criteria

Publications were included if EBV‐SMT involving CNS was mentioned in its content. Included studies should contain patients with confirmed pathologic diagnosis, namely positive stain of EBV‐encoded small RNA (EBER) or EBV DNA detection in samples of smooth muscle tumor. With detection of intratumoral EBV, studies under the diagnosis of EBV‐associated leiomyoma and EBV‐associated leiomyosarcoma were also included. We defined EBV‐SMTs with CNS invasion only if the tumor grew in the brain, spine, skull bone, and vertebrae; but a paraspinal location without craniospinal involvement would be excluded. Moreover, in order to exemplify the effectiveness of treatment, patients with secondary CNS invasion during treatment or follow‐up period would be excluded as well.

### 2.3 Data extraction and documentation

Duplicates among publications were eliminated manually by comparing title, author, and publication date. The remained unique publications were first reviewed by title to select those related to EBV‐associated smooth muscle tumor or leiomyoma/leiomyosarcoma. Further survey through abstract was done subsequently to remove reviews and editorials without new reported cases. Afterward, a thorough full‐text evaluation was conducted to extract reports without definite diagnosis. Subsequently, we performed the documentation of all collected patients with EBV‐SMT and reviewed and combined the data of patients reported in multiple publications case by case. Finally, any patient without initial CNS involvement was extracted. Data extraction was completed under a standardized template. During the review process, all reported data were coded objectively and confirmed by three authors: KWL, YMH, and YTL, respectively. Any unreported or unclear information was recorded as “not available,” in order not to compromise the real situation with a subjective interpretation of data.

### 2.4 Data extraction and statistical analysis

All three first authors (K. W. L, Y. W. H, and Y. T. L) performed the literature screening independently. To extract itemized variables from a literature review, only those with clear statements were documented while variables with ambiguous descriptions or unavailable were recorded as not available. If there was any discordance or data ambiguity, the item would be recorded as “N. A.” (not available). Chi‐square test or Fisher exact test for categorical variables and Wilcoxon rank‐sum test for continuous variables were conducted between the two larger patient groups, that is, HIV infection and POT as listed in Tables 1 and 2. Kaplan–Meier analysis and Log‐rank test were conducted for survival analysis. Only documented treatment modalities and survival outcomes were analyzed as listed in Table 2. A *p* < 0.05 was considered statistically significant.

**TABLE 1:** Summary of 65 patients of EBV‐SMT with CNS invasion in the literature (1997‐2019, including our case)^a^

	HIV/AIDS (*n* = 41, 63.1%)		POT^b^ (*n* = 17, 26.2%)	*p* value*	PID (*n* = 4, 6.2%)	Others^c^ (*n* = 3, 4.6%)	Total (*n* = 65, 100%)
Age (years)	34.02 ± 9.91		31.31 ± 22.71	0.231	11.00 ± 6.21	36.67 ± 34.53	32.07 ± 15.61
Sex: male (%)	21/41 (51.2)		2/17 (11.8)	0.005	1/3 (33.3)	1/3 (33.3)	25/64 (39.1)
Diagnosis by MRI (%)	29/29 (100.0)		7/8 (87.5)^d^	0.216	3/3 (100.0)	3/3 (100)	42/43 (97.7)
Tumor enhanced in contrast MRI (%)	23/29 (79.3)		5/7 (71.4)	0.639	1/3 (33.3)	3/3 (100)	32/42 (76.2)
Latent period (month)^e^	61.54 ± 49.85		43.62 ± 18.72	0.623	11.00 ± 6.21^f^	NA	NA
Tumor size (cm)^g^	3.37 ± 1.40		3.28 ± 1.23	0.857	3	5.60 ± 1.56	3.49 ± 1.41
Multiple tumors at diagnosis (%)	18/36 (50.0)		11/17 (64.7)	0.315	3/4 (75.0)	2/3 (66.6)	34/60 (56.7)
Location of CNS involvement^h^							
Intracranial (%)		30 (73.1)	10 (58.8)	0.282	3 (75.0)	3 (100)	46 (70.8)
Intraspinal (%)		19 (46.3)	6 (35.3)	0.439	1 (25.0)	1 (33.3)	27 (41.5)
Location of involvement out of CNS^i^		13 (31.7)	12 (70.6)	0.006	3 (75.0)	2 (66.7)	30 (46.1)
Head and neck (%)		1/13 (7.7)	2/12 (16.7)	0.593	0	1/2 (50.0)	4/30 (13.3)
Lung (%)		6/13 (46.2)	9/12 (75.0)	0.226	0	0	15/30 (50.0)
Abdomen (%)		5/13 (38.5)	8/12 (66.7)	0.158	2/3 (66.7)	0	15/30 (50.0)
Liver		5	6		2	0	13
Spleen		1	4		1	0	6
Gastrointestinal tract		0	1		1	0	2
Others: peritoneum, mesentery, gall bladder		2	1		0	0	3
Skin, soft tissue, and muscle (%)		5/13 (38.5)	0	0.039	0	0	5/30 (16.7)
Genitourinary tract (%)		4/13 (30.8)	2/12 (16.7)	0.645	1/3 (33.3)	1/2 (50.0)	8/30 (26.7)
Adrenal gland		3	2		1	1	7
Kidney		1	0		0	0	1
Bone^i^ (%)		0	2/12 (16.7)	0.220	0	0	2/30 (6.7)

Abbreviations: AIDS: acquired immunodeficiency syndrome; allo‐PBSCT: allogenic peripheral blood stem cell transplantation; HIV: human immunodeficiency virus; NA: not available; PID: primary immunodeficiency; POT: post‐organ transplantation.

^a^ The presenting data in this table was counted only if it was documented clearly in the literature.

^b^ Type of transplantation: Kidney *n* = 12, Heart *n* = 2, Lung *n* = 1, Bone marrow *n* = 1, allo‐PBSCT *n* = 1.

^c^ All three cases were documented immunocompetent (Age of diagnosis: 8, 27, and 75).

^d^ The cases without MRI studies were surveyed by Computed tomography (CT).

^e^ Time lapse was defined as period of time between underlying disease and EBV‐SMT being diagnosed.

^f^ Latent period of primary immunodeficiency was documented as age of patients.

^g^ The largest diameter of tumor measured was recorded.

^h^ Cases with multiple tumors involved different organ systems will be counted in each category.

^i^ Other than skull and vertebrae, either synchronous or metachronous.

* A statistical analysis was carried out comparing between patients with HIV/AIDS and with POT only due to a combination of >90% of cases by these two categories.

**TABLE 2:** Summary of documented treatment and outcome of the 65 EBV‐SMT patients with CNS invasion (1997‐2019, including our case)^a^

	HIV/AIDS (*n* = 41, 63.1%)	POT^b^ (*n* = 17, 26.2%)	*p* value	PID (*n* = 4, 6.2%)	Others^c^ (*n* = 3, 4.6%)	Total (*n* = 65, 100%)	
Documented treatment options (%)	36/41 (87.7)	15/17 (88.2)		4/4 (100)	3/3 (100)	58/65 (89.2)	
Surgery (%)^b^	36/36 (100.0)	15/15 (100.0)		4/4 (100)	3/3 (100)	58/58 (100.0)	
Total resection (%)	14/36 (38.9)	4/15 (26.7)	0.081	2/4 (50.0)	2/3 (66.7)	22/58 (37.9)	
Subtotal resection (%)	19/36 (51.3)	6/15 (43.8)		1/4 (25.0)	1/3 (33.3)	27/58 (46.6)	
Biopsy only (%)	3/36 (7.7)	5/15 (31.2)		1/4(25.0)	0/3 (0.0)	9/58 (15.5)	
Single tumor	*n* = 16	*n* = 6		*n* = 1	*n* = 1	*n* = 24	
Total resection (%)	9/16 (56.3)	3/6 (50.0)	0.664	1/1 (100)	1/1 (100)	14/24 (58.3)	
Subtotal resection	5	1		0	0	6	
Biopsy	2	2		0	0	4	
Multiple tumors	*n* = 15	*n* = 9		*n* = 3	*n* = 2	*n* = 29	
Total resection	1	1	0.021	1	1	4	
Subtotal resection (%)	14/15 (93.3)	5/9 (55.6)		1/3 (33.3)	1/2 (50.0)	21/29 (72.4)	
Biopsy	0	3		1	0	4	
Adjuvant therapy with or without surgery							
Radiotherapy (%)	13/36 (36.1)^c^	1/15 (6.7)	0.041	0/4 (0.0)	1/3 (33.3)	15/58 (25.9)	
Chemotherapy (%)	3/36 (8.3)	2/15 (20.0)	0.624	1/4 (25.0)	1/3 (33.3)	7/58 (12.1)	
Immune reconstruction (%)^d^	14/36 (38.9)	10/15 (66.7)	0.070	NA	NA	NA	
Documented treatment responses of tumor (%)^e^	20/41 (48.8)	7/17 (41.2)	0.597	3/4 (75.0)	3/3 (100)	33/65 (50.8)	
Complete response (%)	7/20 (35.0)	2/7 (28.6)	0.180	1/3 (33.3)	2/3 (33.3)	12/33 (36.4)	
Partial response (%)	9/20 (45.0)	1/7 (14.3)		1/3 (33.3)	0/3 (0.0)	11/33 (33.3)	
Progressive disease (%)	4/20 (20.0)	4/7 (57.1)		1/3 (33.3)	1/3 (66.7)	10/33 (30.3)	
Local response in surgical group^f^	*n* = 12	*n* = 3		*n* = 2	*n* = 2	*n* = 19	
Local recurrence (%)	1/12 (8.3)	1/3 (33.3)	0.371	1/2 (50.0)	0/2 (0.0)	3/19 (15.8%)	
Documented outcomes (%)	33/41 (80.5)	13/17 (76.5)	1.000	3/4 (75.0)	3/3 (100%)	52/65 (80.0)	
Alive (%)	26/33 (78.8)	5/13 (38.5)	0.014	2/3 (66.7)	2/3 (66.7)	35/52 (67.3)	
ANED (%)	10/26 (38.5)	1/5 (20.0)	0.631	1/2 (50.0)	2/2 (100.0)	14/35 (40.0)	
AWD (%)	16/26 (61.5)	4/5 (80.0)		1/2 (50.0)	0/2 (0.0)	21/35 (60.0)	
Follow‐up period (months, mean ± SD)	22.78 ± 24.68	61.06 ± 47.29	0.023	46.50 ± 36.06	20.50 ± 4.94	30.10 ± 31.33	
Death (%)	7/33 (21.2)	8/13 (61.5)	0.014	1/3 (33.3)	1/3 (33.3)	17/52 (30.9)	
DOD (%)	3/7 (42.9)	2/8 (25.0)	0.631	1/1 (100.0)	0/1(0.0)	6/17 (35.3)	
DOC (%)	4/7 (57.1)	6/8 (75.0)		0/1 (0.0)	1/1 (100.0)	11/17 (64.7)^g^	
Survival time (months, mean ± SD)	16.43 ± 18.04	45.06 ± 47.01	0.385	NA	4.5	30.00 ± 37.57	
Case number	*n* = 29	*n* = 14		NA	*n* = 3	*n* = 48	
6‐month survival (%) (case at risk, censored)	92.8 (23, 4)	85.7 (12, 0)	—	NA	66.7 (2, 0)	89.3 (39, 4)	
1‐year survival (%) (case at risk, censored)	77.8 (15, 9)	70.1 (9, 1)	—	NA	66.7 (2, 0)	76.0 (28, 10)	
2‐year survival (%) (case at risk, censored)	77.8 (10, 14)	70.1 (9, 1)	—	NA	66.7 (1, 1)	76.0 (21, 17)	
5‐year survival (%) (case at risk, censored)	50.0 (2, 20)	60.1 (6, 3)	—	NA	NA	59.6 (9, 26)	
Follow‐up period (months, mean ± SD)	21.30 ± 23.17	52.22 ± 45.83		46.50 ± 36.06	15.17 ± 9.88	30.07 ± 33.13	

Abbreviations: ANED: alive with no evidence of disease; AWD: alive with disease; DOC: dead of other cause; DOD: dead of disease; SD: standard deviation.

^a^ The presented data in this table was counted only if it was documented clearly in the literature.

^b^ Total resection: complete resection of all visible tumors; subtotal resection: unable to resect all tumors with residual; biopsy: only for tissue diagnosis without attempts to remove tumor bulk.

^c^ Twelve out of 13 (92.3%) were arranged after total or subtotal resection.

^d^ Immune reconstruction was considered if antiretroviral agents were started or revised in HIV/AIDS group, and if immunosuppressant were tapered in POT group after diagnosis.

^e^ Treatment response was mostly evaluated by image studies (CT, MRI). *Complete response*: no evidence of residual tumor; *Partial response*: decrease in tumor size; *Progressive disease*: increase in tumor size or new lesions detected.

^f^ We evaluate local recurrence after total resection of a single tumor and complete resection of any one site with multiple tumor involvement.

^g^ Four patients were died of sepsis, other documented causes include CMV retinitis, other intracranial disease, pulmonary embolism, respiratory distress, and cardiac disease.

## 3 RESULTS

### 3.1 Search results

Initial database research revealed 660 publications in total. Additional four studies according to the references of collected publications and one unpublished case of our own were included. First, 236 duplications were extracted by manual screening, and 429 records published were further enrolled (Figure 1). Title and abstract screening subsequently excluded, respectively, 189 and 59 publications which were not related to EBV‐SMT and reviews or editorials without reported cases. Full‐text review among the remaining 181 records resulted in the final inclusion of 45 records which were considered suitable for qualitative analysis, but there was no record suitable for quantitative analysis. The publication year of the records falls within a range from 1997 to 2019.

Among all records, six were case series, and the remaining ones were case reports. Thorough documentation of records was conducted, and 65 patients were enrolled in our final analysis (Table S1).

### 3.2 Demographic data

Table 1 summarizes all 65 patients of EBV‐SMT with CNS invasion in the literature. Patients were classified into four frequently reported and clearly associated predisposing diseases: HIV/acquired immunodeficiency syndrome (AIDS), POT, PID, and others, indicating an immunocompetent status. Among all reported patients with CNS invasion, HIV/AIDS was the most common cause of the immunocompromised status accounted to 63.1% (*n* = 41). On the contrary, only three patients (4.6%) were regarded as immunocompetent status. The mean age at diagnosis was in the third decade, with a second small peak in the first decade (Figure S1). Patients with POT were significantly less likely to be male (*n* = 2, 11.8%), while 51.2% (*n* = 21) of patients with HIV/AIDS were male (*p* = 0.005). Radiologically, 76% of reported patients underwent MRI had contrast enhancement in their tumors. The mean latent period for the diagnosis of EBV‐SMT was around 4 years after patients’ immune system were compromised in HIV/AIDS and POT groups. The average tumor size was 3.3 cm in immunocompromised patients and 5.6 cm in immunocompetent patients. Overall, 56.7% (*n* = 34) of the patients were reported to have multiple EBV‐SMT tumors at diagnosis. Around 70% (*n* = 46) of patients had intracranial invasion, while 41.5% (*n* = 27) of patients had intraspinal invasion. Forty‐six percent (*n* = 30) of patients presented with extra‐CNS involvement concomitantly: lung (50%, *n* = 15), abdomen (50%, *n* = 15), and genitourinary tract (26.7%, *n* = 8) being the top three locations. Patients with POT had significantly higher rate of extra‐CNS involvement (*p* = 0.006). Compared to patients with POT, patients with HIV/AIDS had a higher proportion of skin and soft tissue invasion (*p* = 0.039), while a trend toward higher proportion of abdominal invasion was noted in patients with POT (*p* = 0.158).

### 3.3 Therapeutic strategies and outcomes

Table 2 summarizes the therapeutic options, treatment responses, and outcomes. Notably, 89.2% of reported cases (*n* = 58/65) had their treatment options recorded; while 50.8% of treatment responses (*n* = 33/65) and 80.0% of final outcomes (*n* = 52/65) were documented. Among reported treatment options for EBV‐SMT involving CNS, all patients underwent surgical resection. Regarding extent of resection (EOR), 22 patients (37.9%) and 27 patients (46.6%) underwent total and subtotal resections, respectively, giving a total of 49 patients (84.5%) with at least subtotal resection. For patients with single tumor (*n* = 24), 58.3% (*n* = 14) of whom underwent total resection; while 72.4% of patients (*n* = 21) with multiple tumors had subtotal resection. There was a trend toward higher EOR among patients with HIV/AIDS compared to those with POT (*p* = 0.081), the difference was most likely to result from a significant higher rate of EOR for those with multiple tumors (*p* = 0.021).

After surgery, radiotherapy, chemotherapy, and immune reconstruction were three most widely adopted adjuvant therapies. Choice of adjuvant radiotherapy was statistically different between patients with HIV/AIDS and POT: among them, a significantly higher proportion of patients with HIV/AIDS than patients with POT received adjuvant therapy (36.1% vs. 6.7%, *p* = 0.041). On the contrary, patients with POT had a trend toward higher rate of adjusting medications for immune reconstruction than patients with HIV/AIDS (66.7% vs. 38.9%, *p* = 0.070). Adjuvant chemotherapy was seldomly carried out in both groups (8.3% vs. 20%, *p* = 0.624).

**FIGURE 2:**
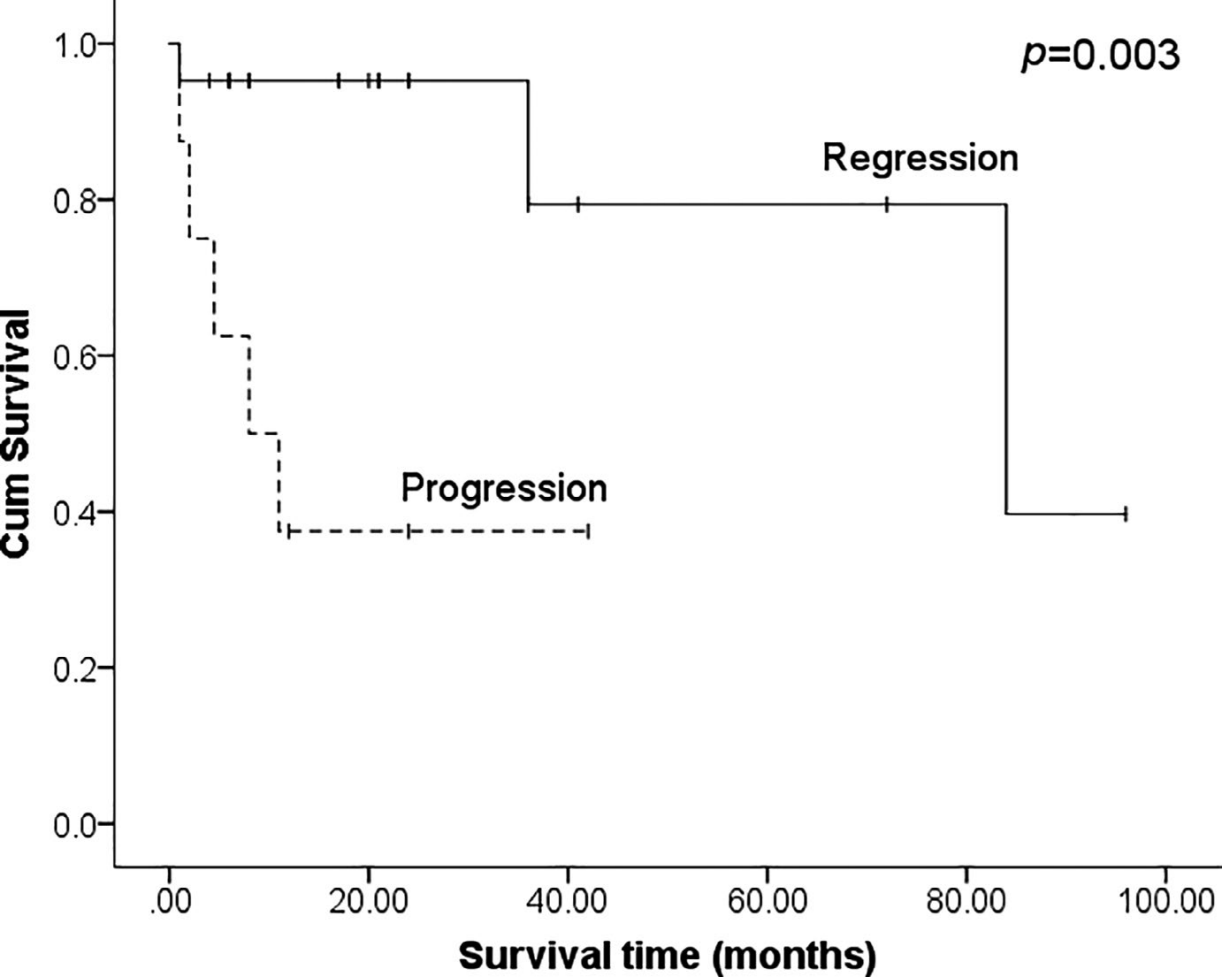
Overall survival between locally regressive and aggressive tumors

For treatment response of tumors, nearly equal complete response (CR, 36.4%), partial response (PR, 33.3%), and progressive disease (PD, 30.3%) were found and there was no difference between the HIV/AIDS and POT groups (Table 2). Specifically, only three patients (15.8%) had documented local recurrence after surgical resection. For the evaluation of local response, a significant survival benefit could be observed when comparing patients with regression of local tumor (CR and PR) to patients with PD (*p* = 0.003, Figure 2), indicating a locally regression of tumor predicts a better overall survival than a locally progressive one.

For patient outcomes, nearly one‐third of patients were dead with a mean survival time of 30 months and nearly two‐thirds of them died for other causes; on the contrary, 60% of the survived patients, lived with the disease (AWD, Table 2). In subgroup analysis, 78.8% (*n* = 26) of patients with HIV/AIDS were alive, and 38.5% (*n* = 10) of them were alive with no evidence of disease (ANED) at a mean follow‐up period of 22.8 months. On the contrary, 38.5% (*n* = 5) of patients with POT were alive, and 20% (*n* = 1) of them were ANED, with a mean follow‐up period of 61.1 months (*p* = 0.023). Overall, 67.3% (*n* = 35) of documented patients were alive, and 40% (*n* = 14) of them were ANED, with a mean follow‐up period of 30.1 months. Notably, a significant survival difference was found between patients with HIV/AIDS and those with POT (*p* = 0.014), however, the follow‐up period was also significantly different between the two groups (22.78 ± 24.68 vs. 61.06 ± 47.29 months, *p* = 0.023). Finally, despite heterogeneous follow‐up periods, 6‐month, 1‐year, 2‐year, and 5‐year survivals were 89.3%, 76%, 76%, and 59.6% in the 48 reported patients, respectively, with a mean follow‐up period of 30.07 ± 33.13 months.

### 3.4 Role of surgical resection

**FIGURE 3:**
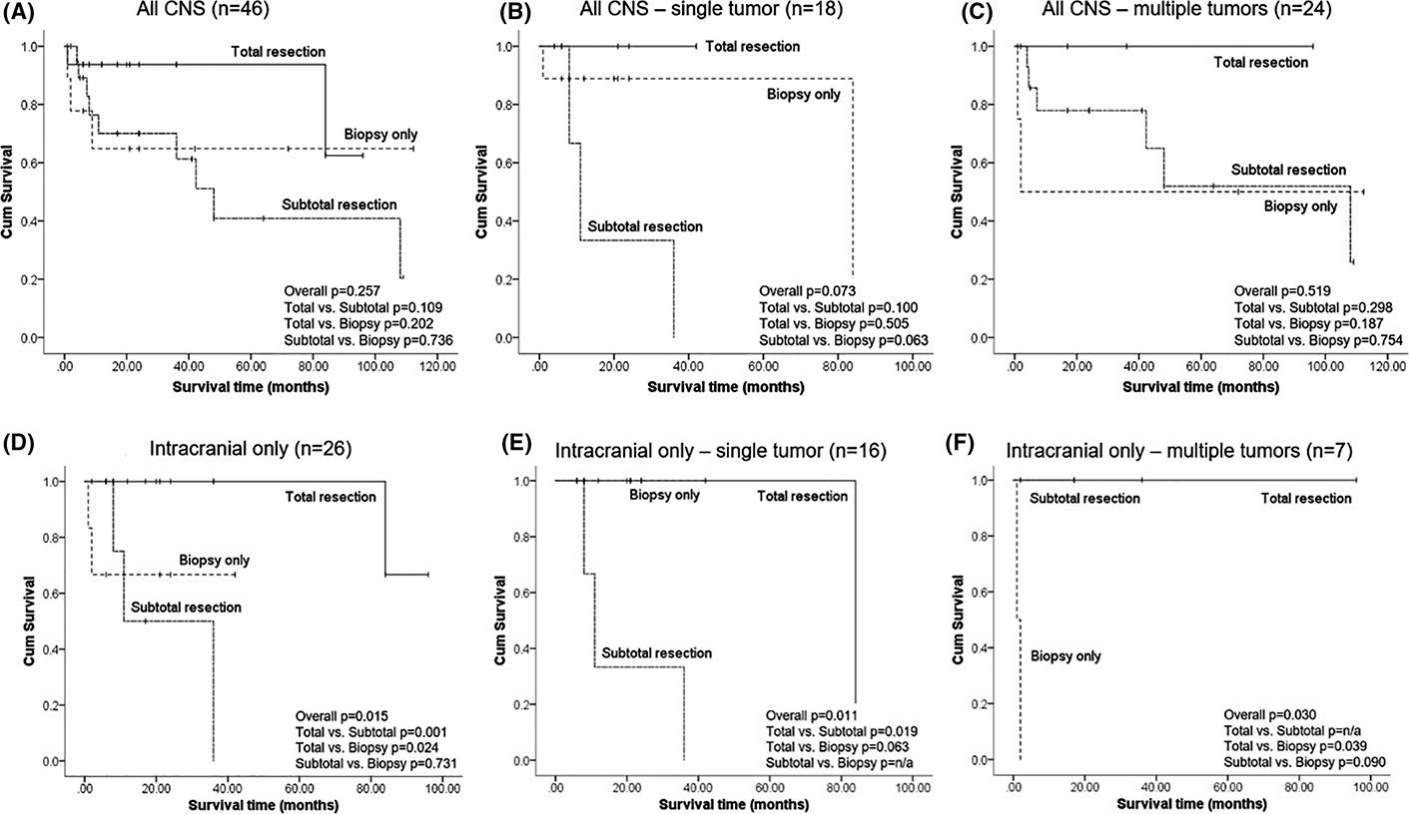
Survival analysis of different EOR in all CNS invasion cases (A); in cases with single tumor (B); in cases with multiple tumors (C); and in all cases with intracranial invasion (D); in cases with single intracranial tumor (E); and in cases with intracranial multiple tumors (F)

According to our analysis, surgical resection, excluding biopsy, was most commonly conducted (84.5%) in patients with CNS invasion. Since nearly all patients received surgical resection for decompression or diagnosis or both, an EOR analysis on treatment response of tumors and patients’ survival were thoroughly conducted. For locally tumor control, there was a significantly lower rate of progression of tumor when comparing total resection to non‐total resection (*p* = 0.0495, Table S2). However, there was no survival difference among patients who underwent total resection, subtotal resection or biopsy (*p* = 0.257, Figure 3A). The lack of impact of EOR on survival remained true in further analysis of subgroups of single tumor and multiple tumors (*p* = 0.073 and 0.519, respectively, Figure 3B,C). Interestingly, if only patients with intracranial tumors (n = 26, Figure 3D‐E) were taken into account, a significantly better survival was found in those underwent total resection than those having non‐total resection (*p* = 0.015, Figure 3D). The survival benefit toward higher EOR in patients with intracranial invasion remained statistically significant by subgroup analysis of tumor multiplicity. (*p* = 0.011 and 0.03, respectively, Figure 3E and F).

## 4 DISCUSSION

Immunodeficiency is considered as the principal factor of the pathogenesis of EBV‐SMT.^32^ HIV/AIDS, chronic usage of immunosuppressant in patients with POT, and PID were reported to be the three main causes of immunodeficiency leading to EBV‐SMT.^11^, ^33^ Among all 65 cases with CNS invasion, 96% of patients were immunodeficient. One of the three reported immunocompetent cases bore the highest age of diagnosis among all cases (Table S1); however, whether aging could play a role in the pathogenesis of EBV‐SMT remains uncertain. According to a high propensity of cases with EBV‐SMT were under immunodeficient status, an exhaustively review on immune status should be conducted for all patients diagnosed with EBV‐SMT. Interestingly, comparing to the patients with other underlying modalities, a significant predilection of CNS involvement for HIV/AIDS patients was discovered during our review (44/109 vs. 26/116 cases in patients underwent POT, *p* < 0.001, data not shown), which has been previously hypothesized.^6^, ^17^ This finding could possibly explain the reason why patients with POT had higher propensity for extra‐CNS involvement in our review, since CNS invasion could be metachronously developed under prolonged immunodeficiency with undiagnosed extra‐CNS tumor.

We found a female predilection for CNS invasion in POT group, while a previous review of all locations of EBV‐SMT in POT patients showed no gender predilection.^34^ A comparison of demographic data including patient age, type of transplantation, or latent period between genders to the previous study^34^ reveals no difference between both groups. Such results exclude the possibility that a female recipient lives longer than a male recipient to develop EBV‐SMT, which is in line with several reports regarding the impact of gender on survival of POT patients.^35^, ^36^, ^37^ Therefore, a possible higher risk for female POT patients to develop CNS EBV‐SMT could be hypothesized; however, the patho‐mechanism for this observation remains to be answered.

EBV‐SMT has been discovered in different locations in the CNS, including cerebral lobes, basal ganglia, cerebral sinuses, cerebellum, and intraspinal regions from cervical to sacrum. Interestingly, among the aforementioned locations, the intracranial parasellar region including cavernous sinus and sphenoid area were particularly reported several times (Table S3). It could possibly be explained by the hypervascularity within cavernous sinus, based on the hypothesis of EBV‐SMT’s vascular smooth muscle cell origin.^17^, ^34^, ^38^

As for concomitant EBV‐SMTs outside the CNS, a trend toward higher abdominal invasion has been shown in POT patients; on the contrary, it was more common for patients with HIV/AIDS to have concomitant soft tissue invasion, which is in agreement with the previous two reviews. Jonigk et al have shown that liver and lung were the two most common locations of EBV‐SMTs in POT patients,^34^ which were possibly attributable to their rich vascularity based on the pathophysiology of EBV‐SMT^39^; while CNS and soft tissue of HIV/AIDS patients were more frequently documented in Purgina's study.^17^ This location distribution coincides with lymphoproliferative disorders sharing similar pathophysiology to EBV‐SMT,^32^ namely posttransplant lymphoproliferative disorders (PTLD) in POT patients and non‐Hodgkin lymphomas in AIDS patients, wherein GI tract and CNS were more commonly involved, respectively.^40^, ^41^ Moreover, every POT patient in our review with liver involvement as synchronous extra‐CNS tumor had concomitant lung involvement, which makes up 66.7% of lung EBV‐SMTs (6 out of 9). To our knowledge, this scenario has not been mentioned in previous publications of EBV‐SMT, and was also infrequent for other common POT tumors^42^ and it hardly could be explained just by the effect of impaired immunosurveillance, preexisting risk factors of de novo cancer in transplanted organ, or target organ of EBV infection.^43^ Despite requiring further explanation, a high tendency toward concomitant liver and lung invasion in POT patients should be kept in mind while surveying synchronous extra‐CNS EBV‐SMTs.

Owing to the untraceable period in HIV/AIDS group between HIV infection and definite medical diagnosis of AIDS, documented latent period varies widely, ranging from contemporary to 204 months after diagnosis of AIDS.^30^, ^38^ Conversely, in POT patients, a latent period of around 4 years was more reliable, which was similar to the previous report.^34^ Last but not least, cases with multiple tumors were frequently documented, either synchronously or metachronously, which was identical to previous publications.^10^, ^11^ Despite its frequency, metastasis was considered not the cause of multiple EBV‐SMTs.^14^

To treat EBV‐SMT with CNS invasion, a variety of modalities including surgical resection, immune reconstruction, radiotherapy, and chemotherapy were attempted. Several publications indicate that immune reconstruction by starting antiviral treatment in HIV/AIDS patients and adjusting immunosuppressant in POT patients is a fundamental strategy for patients with EBV‐SMT; meanwhile, it could also prevent opportunistic infections, which hold a great survival risk to these patients.^11^, ^17^, ^33^ The difference between HIV/AIDS and POT patients on performing immune reconstruction was possibly attributable not only to the complexity and low predictability on medication adjustment in HIV/AIDS compared to POT group, but also to poor patient compliance mentioned in several reports.^30^, ^44^ A higher proportion of choices of radiotherapy as adjuvant treatment in patients with HIV/AIDS than patients with POT were found, despite the tumor size and EOR were similar in both groups (Table 2). One possible explanation might be related to the treatment philosophy of patients with HIV/AIDS and patient with POT, meaning an immunocompromised status could not easily be reversed by adjusting antiviral medications in the former, while tapering immunosuppressants theoretically resume part of immune functions in the latter group. To selecting chemotherapeutic regimens, a response to bevacizumab in multiple EBV‐SMTs with pathologic features similar to malignant pericytoma has been reported,^45^ while other attempts with temozolomide or combination of vincristine, doxorubicin, and cyclophosphamide showed no favorable outcome.^21^, ^46^ In addition, Sirolimus has been suggested as a substitute immunosuppressant for POT patients according to the activation of mTOR/Akt pathway in EBV‐SMTs.^18^, ^32^ Further research is required to investigate the effectiveness and selection of adjuvant therapy after initial surgical resection for CNS EBV‐SMT.

In the light of previous publications,^5^, ^11^, ^17^ EBV‐SMT has been thought of as an indolent tumor, featuring slow growing rate and seldom tumor‐related death. In this review on patients with CNS invasion, outcome was comparable. Nearly two‐thirds of patients were alive, and two‐thirds of whom were AWD. For the one‐third dead patients, only one‐third of them were DOD. Besides, there was no difference of survival on tumor multiplicity (*p* = 0.746, data not shown). Despite concomitant with multiple underlying medical conditions, 1‐year survival rate of 76.0% and 5‐year survival rate of 59.6% have been concluded. Although a trend of higher survival has been shown in HIV/AIDS patients, a significant shorter follow‐up period was noted. This justified nearly identical 1‐year, 2‐year, and 5‐year survivals in patients with HIV/AIDS and those with POT (Table 2).

Finally, we interrogated the role of surgery in treating patients with CNS EBV‐SMT. The observation of a local regression of tumor predicting a better overall survival than a locally progressive one may reflect the fact that a local control of tumor is resulted from an improved immune function. The survival benefit of total resection for those with intracranial invasion, instead of all, CNS EBV‐SMT may be due to only intracranial tumor causing mass effect or cranial nerve dysfunction hampers patients’ survival. Therefore, it is understandable for patients who underwent higher EOR had better survival. Besides, the impact of survival by greater EOR seems to be more evident for single tumor than multiple tumors. A further support as mentioned earlier indicates that one‐third of the tumors progressively enlarged after treatment, which further threatens prognosis if a tumor locates intracranially.

The main limitation of this study is the heterogeneous data acquirement from a retrospective approach, and the poor patient consistency, diverse treatment modalities, and varying follow‐up period may influence the quality and possibility of statistical analysis. Also, publication and selection bias are inherent deficits in this retrospective review and care must be taken for interpreting the role of surgical intervention since there is no control on direct comparison. However, a prospective study should never be conducted for such a low incidence tumor type. Therefore, despite these flaws, with stringent inclusion and exclusion criteria during data collection, the analysis of clinically relevant questions has been made possible. We believe this review can provide valuable epidemiologic and demographic references and therapeutic and prognostic information for patients with EBV‐SMT who have CNS invasion.

## 5 CONCLUSION

In summation, over 95% of reported EBV‐SMT patients with CNS invasion were immunocompromised, while HIV/AIDS and POT being the most commonly associated underlying causes (90%). EBV‐SMTs in HIV/AIDS patients have a predilection for CNS involvement than in POT patients. Although commonly present with multiple tumors, there is no survival difference among different predisposing diseases or tumor multiplicity. Immune reconstruction should be considered for all patients since the pathogenesis of EBV‐SMT is considered to be strongly associated with immunocompromised status. In other words, any patient diagnosed with EBV‐SMT without a known HIV/AIDS, chronic immunosuppressant usage or PID history, a thorough survey of immune function should be conducted. A surgical resection remains the first‐line treatment in combination with various adjuvant modalities. Patients with EBV‐SMT invading CNS may benefit from total resection for tumor control, however, may not prolong survival. Surgery should be reserved for diagnosis requirement or relief of focal mass effect. A total resection may offer survival benefit if the tumor is symptomatic and has intracranial invasion.

## AUTHOR CONTRIBUTIONS

K.‐T. C. developed the hypothesis and designed the paradigm. K.‐W. L., Y.‐W. H., and Y.‐T. L. collected data, reviewed literatures, and wrote the manuscript. K.‐T. C., K.‐W. L., Y.‐W. H., and Y.‐T. L. analyzed data together and revised the draft. There was no conflict of interests for all authors. All analytic results and supplementary materials are available from the manuscript.

## Supporting information

Fig S1

Table S1

Table S2

Table S3
