# Supplementary figures and images for: Role of surgery in treating epstein‐barr virus‐associated smooth muscle tumor (EBV‐SMT) with central nervous system invasion: A systemic review from 1997 to 2019
Source: Cancer Med. 2021 Feb 11;10(5):1473–84. doi: 10.1002/cam4.3770 (PMC7940242; doi:10.1002/cam4.3770)

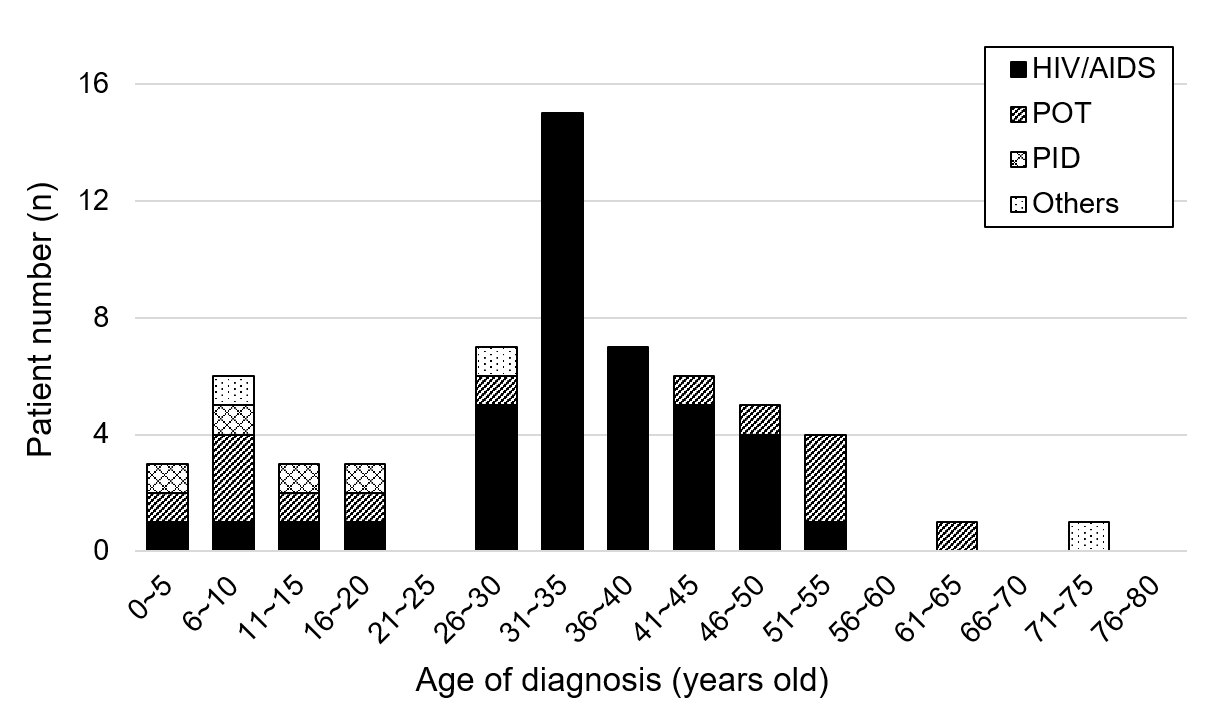

Supplement: Supplementary file 1 — Fig S1 [file CAM4-10-1473-s001.tif]
