# Supplementary material for: Role of surgery in treating epstein‐barr virus‐associated smooth muscle tumor (EBV‐SMT) with central nervous system invasion: A systemic review from 1997 to 2019
Source: Cancer Med. 2021 Feb 11;10(5):1473–84. doi: 10.1002/cam4.3770 (PMC7940242; doi:10.1002/cam4.3770)
Supplement: Supplementary file 2 — Table S1 [file CAM4-10-1473-s003.docx]

| Case No. | Author  (publication year) | Age (years), sex | Underlying condition | |  | CNS invasion | | Extra-CNS location(s) | Treatment | | | Outcome,  f/u duration (months) |
| --- | --- | --- | --- | --- | --- | --- | --- | --- | --- | --- | --- | --- |
|  |  |  | Disease category | Latent period (months) |  | Category | Tumor location(s) |  | Surgical option | Adjuvant therapy | Response |  |
| 1 | Jenson (1997)/Jenson (1999) | 29, F | HIV/AIDS | 24 |  | S | T spine |  | NA | NA | NA | NA |
| 2 | Mierau (1997)/ Kleinschmidt-Demasters(1998) | 14, F | PID (CVID) | NA |  | C | Left temporal lobe |  | Total |  | CR | ANED, 21 |
| 3 | Morgello (1997) | 35, M | HIV/AIDS | NA |  | S | Right C6 spinal dura |  | Total |  | CR | DOC, 1 |
| 4 | Kleinschmidt-DeMasters(1998) | 34, F | HIV/AIDS | 48 |  | C | Right cavernous sinus |  | Subtotal |  | PD | DOC, 8 |
| 5 | Litofsky (1998) | 50, M | HIV/AIDS | 72 |  | C | Right occipital brain surface |  | Total |  | CR | ANED, 8 |
| 6 | Blumenthal (1999) | 43, M | HIV/AIDS | 144 |  | C | Cavernous sinus |  | Biopsy | CT | NA | AWD, 24 |
| 7 | Brown (1999) | 34, F | HIV/AIDS | NA |  | C | Left of pontine cistern |  | Total |  | PD | AWD, 12 |
| 8 | Karpinski (1999) | 26, M | HIV/AIDS | NA |  | C+S | Left sphenoid wing,  C2-3 spinal intradura |  | NA | NA | NA | NA |
| 9 | Ritter (2000) | 5, F | HIV/AIDS | 42 |  | C | Left cavernous sinus |  | Subtotal |  | PD | AWD, NA |
| 10 | Ritter (2000) | 35, F | HIV/AIDS | 96 |  | S | T3, T4 spine |  | Total | RT | NA | NA |
| 11 | Collins (2001) | 7, F | POT (Heart) | 60 |  | C | Intracranial epidura |  | Biopsy | ISA | PD | AWD, 42 |
| 12 | Boudjemaa (2004) | 5, M | POT (Renal) | 36 |  | C | Right temporal lobe | Liver, lung, spleen, mesentery | Biopsy | CT, ISA | PD | DOD, 2 |
| 13 | Kumar (2004) | 10, NA | HIV/AIDS | 120 |  | C | Basal ganglia |  | Total |  | CR | ANED, 6 |
| 14 | Zevallos-Giampietri (2004) | 29, M | HIV/AIDS | 36 |  | C | Left extra-axial parasella |  | Subtotal | RT | PR | AWD, 6 |
| 15 | Suankratay (2005)/ Issarachaikul (2014) | 43, F | HIV/AIDS | 24 |  | C+S | Medial tentorium cerebelli, T3-6 spinal epidura |  | Subtotal | ART, RT | NA | DOC, 4 |
| 16 | Suankratay (2005)/ Issarachaikul (2014) | 34, F | HIV/AIDS | 96 |  | S | T3-5, T9-11, L1-2 spinal epidura | Adrenal gl. | Subtotal | ART, RT | PD | AWD, 24 |
| 17 | Suankratay (2005)/ Issarachaikul (2014) | 49, F | HIV/AIDS | 48 |  | C+S | Medial tentorium cerebelli, frontal lobe | Orbit | Subtotal | RT | NA | DOD, 48 |
| 18 | Suankratay (2005)/ Issarachaikul (2014) | 34, F | HIV/AIDS | 60 |  | C | Medial tentorium cerebelli |  | Total | RT | CR | ANED, 8 |
| 19 | Suankratay (2005)/ Issarachaikul (2014) | 31, F | HIV/AIDS | 12 |  | C+S | Right cerebellopontine angle, T7-8, T10-12, L4 spinal epidura |  | Subtotal | RT | NA | AWD, 5 |
| 20 | Suankratay (2005)/ Issarachaikul (2014) | 35, M | HIV/AIDS | 0 |  | C | Parietal, frontal parasagittal |  | Total | ART | CR | ANED, 96 |
| 21 | Deyrup (2006) | 38, M | HIV/AIDS | 114 |  | S | Spinal cord | Liver, lung, gallbladder | NA | NA | NA | DOD, 9 |
| 22 | Deyrup (2006) | 50, F | POT (Renal) | 30 |  | NA | Epidura | Lung, vocal cord | NA | NA | NA | DOC, 105 |
| 23 | Deyrup (2006) | 41, F | HIV/AIDS | 24 |  | NA | Epidural |  | NA | NA | NA | AWD, 19 |
| 24 | Chaves (2007) | 19, F | POT (Lung) | 15 |  | C | Parasagittal occipital lobe |  | Subtotal | ISA, RT | PD | DOC, 11 |
| 25 | Gallien (2008)/ Calderaro(2008) | 35, M | HIV/AIDS | 19 |  | C+S | Temporoparietal lobe, Sphenoid bone, Spinal dura | Liver | Subtotal | ART | PR | AWD, 41 |
| 26 | Zevgaridis (2009) | 45, F | POT (Renal) | 48 |  | C | Left anterior temporal lobe |  | Total |  | CR | DOC, 84 |
| 27 | Ong (2009) | 52, F | POT (Renal) | 24 |  | S | Spine | Liver, lung, vocal cord | Subtotal | ISA | NA | DOC, 108 |
| 28 | Ong (2009) | 27, M | POT (Renal) | 84 |  | S | Spine | Liver, lung | Subtotal | ISA | NA | Alive, 64 |
| 29 | Gupta (2010) | 17, F | HIV/AIDS | NA |  | C | Right paracentral prepontine area |  | Biopsy | CT, RT | PR | AWD, 21 |
| 30 | Sivendran (2011) | 43, M | HIV/AIDS | NA |  | C | Frontal lobe |  | Total | ART | CR | ANED, 20 |
| 31 | Ibebuike (2012) | 37, M | HIV/AIDS | NA |  | C | Frontal lobe |  | Subtotal | ART | PR | DOC, 36 |
| 32 | Jonigk (2012) | 13, F | POT  (Bone marrow) | 52 |  | C | Cerebral sinus | Spleen | NA | NA | NA | NA |
| 33 | Takei (2013) | 27, M | No immunodef. | NA |  | C | Frontal lobe |  | Total | CT, RT | CR | ANED, 24 |
| 34 | Lohan (2013) | 37, M | HIV/AIDS | NA |  | C+S | Greater wing of sphenoid, clivus, T6 spinal epidural | Spinal muscle | Subtotal |  | PR | AWD, NA |
| 35 | Lohan (2013) | 55, F | POT (Renal) | 36 |  | C | Lesser wing of sphenoid |  | Total |  | NA | NA |
| 36 | Tan (2013) | NA | POT (Renal) | NA |  | S | Epidural of T5-6 spine | Lung | Subtotal | ISA | NA | DOC, 42.3 |
| 37 | Tan (2013) | NA | POT (Renal) | NA |  | S | Spine | Femur head | Subtotal |  | NA | DOC, 7.2 |
| 38 | Tan (2013) | NA | POT (Renal) | NA |  | S | Spine | Liver, lung | Biopsy | ISA | NA | AWD, 112.3 |
| 39 | Tan (2013) | NA | POT (Renal) | NA |  | S | Sacral area | Liver, lung, adrenal gl., spleen | Subtotal | ISA | NA | AWD, 109 |
| 40 | Petersson (2013) | 55, F | POT (Renal) | 48 |  | C | Frontotemporal lobe |  | Total |  | NA | NA |
| 41 | Petersson (2013) | 37, M | HIV/AIDS | 8 |  | C | Sphenoid bone, clivus, parafalcine region, frontal convexity | Liver, lung, spleen, adrenal gl., gluteus | Subtotal |  | NA | NA |
| 42 | Kazmi (2014) | 8, F | POT (Renal) | 60 |  | C | Frontal, frontotemporal lobe | Adrenal gl., intestine | Total | ISA | CR | ANED, 36 |
| 43 | Issarachaikul (2014) | 32, F | HIV/AIDS | 120 |  | C | Cranial epidura |  | Total | RT | NA | ANED, 84 |
| 44 | Issarachaikul (2014) | 33, F | HIV/AIDS | 60 |  | C+S | Cranial and spinal epidura | Lung | Subtotal | ART, RT | NA | ANED, 48 |
| 45 | Issarachaikul (2014) | 31, F | HIV/AIDS | NA |  | S | Spinal epidura |  | Total | ART | NA | ANED, 12 |
| 46 | Issarachaikul (2014) | 36, F | HIV/AIDS | NA |  | S | Spinal epidura |  | Biopsy |  | NA | DOD, 9 |
| 47 | Issarachaikul (2014) | 29, F | HIV/AIDS | 24 |  | C | Cranial epidura |  | Total | RT | NA | AWD, 6 |
| 48 | Issarachaikul (2014) | 36, F | HIV/AIDS | 84 |  | C | Cranial epidura | Lung, kidney | Subtotal | ART | NA | AWD, 2 |
| 49 | Issarachaikul (2014) | 39, M | HIV/AIDS | 12 |  | C | Cranial epidura |  | Total | ART | NA | ANED, 36 |
| 50 | Wilaisakditipakorn (2015) | 11, M | HIV/AIDS | 120 |  | S | C3–4 spinal intradural-extramedulla |  | Subtotal | ART | PR | AWD, 4 |
| 51 | Raheja (2016) | 65, F | POT (alloPBSCT) | 24 |  | C | Cavernous sinus |  | Biopsy | ISA | PR | AWD, 6 |
| 52 | Parta (2017) | 18, M | PID  (GATA2 def.) | NA |  | S | T, L Spine | Liver, spleen | Biopsy |  | PR | AWD, 72 |
| 53 | Garg (2017) | 8, F | PID (Unknown) | NA |  | C | Left jugular to scull base | Adrenal gl. | Total |  | NA | NA |
| 54 | Munjal (2017) | 33, F | HIV/AIDS | NA |  | S | T10-L1 spinal extradura | Liver, lung | Subtotal | ART | PR | AWD, 1 |
| 55 | Szymanski (2017) | 31, M | HIV/AIDS | NA |  | C | Parietal-occipital, frontal lobe, temporal lobe, cerebellum | Liver, lung, adrenal gl. | Subtotal | ART, CT, RT | PR | AWD, 17 |
| 56 | Pather (2017) | 53, M | HIV/AIDS | NA |  | C | Intracranial |  | Total |  | CR | ANED |
| 57 | Schober (2017) | 4, NA | PID  (CARMIL2 def.) | NA |  | C | Medulla oblongata | Liver, intestine, colon | Subtotal | CT | PD | DOD |
| 58 | Derakhshan (2017) | 44, M | HIV/AIDS | NA |  | C | Frontal lobe |  | Total |  | NA | NA |
| 59 | Cheng (2017) | 42, M | HIV/AIDS | 72 |  | C+S | Cerebellum, L1-L3/S1-S3 spine |  | Subtotal | ART | NA | NA |
| 60 | Chiu (2018) | 35, M | HIV/AIDS | NA |  | C | Near foramen mangum | Chest wall | Subtotal |  | NA | NA |
| 61 | Thammachantha (2018) | 26, M | HIV/AIDS | 24 |  | S | C6-7 spinal intradural-extramedulla |  | NA | NA | NA | NA |
| 62 | Brown (2018) | 8, F | No immunodef. | NA |  | C | Skull base | Adrenal gl. | Total |  | CR | ANED, 17 |
| 63 | Stubbins (2019) | 6, F | POT (Heart) | 50 |  | C | Brain | Liver, lung, spleen | Biopsy | CT | PD | DOD, 1 |
| 64 | Wujanto (2019) | 43, M | HIV/AIDS | 16 |  | C+S | Posterior fossa, T6/T9/L3/S2/S3 spine |  | Subtotal | RT | PR | AWD, 24 |
| 65 | Lau (2019) | 75, F | No immunodef. | NA |  | C+S | C1-C2 spine, post. scull base | Tonsil | Subtotal | ISA | PD | DOC, 4.5 |

NA, non-available; M, male; F, female; HIV, human immunodeficiency virus; AIDS, acquired immune deficiency syndrome; PID, primary immunodeficiency; CVID, common variable immunodeficiency; POT, post-organ transplantation; alloPBSCT, allogeneic peripheral blood stem cell transplantation; C, intracranial; S, intraspinal; ART, antiretroviral therapy; CT, chemotherapy; ISA, immunosuppressant adjustment; RT, radiotherapy; CR, complete response; PD, progressive disease; PR, partial response; ANED, alive with no evidence of disease; AWD, alive with disease; DOC, dead of other cause; DOD, dead of disease; f/u, follow-up

Supplementary table 1. Summary of all reported EBV-SMT cases with CNS invasion in the literature (n=65)
