# Supplementary material for: Role of surgery in treating epstein‐barr virus‐associated smooth muscle tumor (EBV‐SMT) with central nervous system invasion: A systemic review from 1997 to 2019
Source: Cancer Med. 2021 Feb 11;10(5):1473–84. doi: 10.1002/cam4.3770 (PMC7940242; doi:10.1002/cam4.3770)
Supplement: Supplementary file 3 — Table S2 [file CAM4-10-1473-s002.docx]

**Supplementary Table 2-1. Treatment outcome after surgery of all documented cases (*n*=33)***

| Surgery type | Treatment outcome^a^ | | |  |
| --- | --- | --- | --- | --- |
|  | Progression (%) | Regression (%) | Total (%) | *p-value*^b^ |
| Total resection | 1 (7.7) | 12 (92.3) | 13 (100) | 0.0495 |
| Non-total resection^c^ | 9 (45.0) | 11 (55.0) | 20 (100) |  |
| Total | 10 (30.3) | 23 (69.7) | 33 (100) |  |

*Due to the limitation of literature review, only 33 cases underwent surgical resection reported clear outcome were included in our record. This composed of 44% (33/75) of all reported patients with EBV-SMT.

^a^ Progression: Increase in size of residual tumor or new lesion discovered

Regression: Decrease in size of residual tumor including complete removal with no recurrence

^b^ Result is based on Fisher’s exact test

^c^ Including subtotal resection and biopsy

**Supplementary Table 2-2. Treatment outcome after surgery of all documented cases (*n*=27)***

| Surgery type | Treatment outcome^a^ | | |  |
| --- | --- | --- | --- | --- |
|  | Progression (%) | Regression (%) | Total (%) | *p-value*^b^ |
| Total resection | 1 (7.7) | 12 (92.3) | 13 (100) | 0.077 |
| Subtotal resection | 6 (42.9) | 8 (57.1) | 14 (100) |  |
| Total | 7 (25.9) | 20 (74.1) | 27 (100) |  |

*A subgroup analysis including only total and subtotal resection (n=27), which was 81.8% (27/33) of all documented surgically treated cases.

^a^ Progression: Increase in size of residual tumor or new lesion discovered

Regression: Decrease in size of residual tumor including complete removal with no recurrence

^b^ Result is based on Fisher’s exact test
