# Supplementary material for: Role of surgery in treating epstein‐barr virus‐associated smooth muscle tumor (EBV‐SMT) with central nervous system invasion: A systemic review from 1997 to 2019
Source: Cancer Med. 2021 Feb 11;10(5):1473–84. doi: 10.1002/cam4.3770 (PMC7940242; doi:10.1002/cam4.3770)
Supplement: Supplementary file 4 — Table S3 [file CAM4-10-1473-s004.docx]

**Supplementary table 3. Summary of locations of CNS invasion of all EBV-SMT (Intracranial n=46 , Intraspinal n=27)**

|  | HIV/AIDS | POT | PID and Others |
| --- | --- | --- | --- |
| Intracranial  (n=46) | Cavernous sinus (n=3)  Sphenoid (n=3) *(sphenoid wing, n=2)*  Clivus (n=2)  Parasellar (n=1)  Supratentorial  F lobe (n=6) *(frontal convexity, n=1, dural based, n=2)*  F-P lobe *(parasagittal, n=1)*  T-P lobe (n=1)  T lobe (n=2) *(with sphenoid bone erosion, n=1)*  P-O lobe (n=1)  O lobe *(surface, n=1)*  Basal ganglia (n=1)  Parafalcine area (n=1)  Infratentorial  Tentorium cerebelli (n=2)  Cerebellum(n=2)  Parapontine (n=3)  *(pontine cistern, prepontine area, CP angle)*  Near foramen magnum (n=1)  Non-specific  Intracranial (n=1)  Cranial epidural (n=5) | Cerebral sinus (n=1)  Cavernous sinus (n=1)  Sphenoid *(lesser wing, n=1)*  Supratentorial  F lobe (n=1)  F-T lobe (n=2)  T lobe (n=2)  O lobe *(prasagittal, n=1)*  Non-specific  Intracranial epidural (n=1)  Brain (n=1) | F lobe *(invading corpus callosum, n=1)*  T lobe (n=1)  Skull base (n=3)  *(jugular foramen, n=1, posterior skull base, n=1)*  Medulla oblongata (n=1) |
| Intraspinal  (n=27) | C spine (n=5) *(C1-6, C2-3, C3-4, C6, C6-7)*  T spine (n=10)  *(T2(n=2), T3-4, T3-5, T3-6, T6, T7-8, T9-11, T10-12)*  L spine (n=4) *(L1-2, L1-3, L3, L4)*  S spine *(S1-S3)*  T6/T9/L3/S2/S3  T10-L1  Non-specific  Spinal cord (n=1)  Spinal epidural (n=3) | T spine *(T5-6, n=1)*  Sacral region (n=1)  Non-specific  Spine (n=4) | C spine *(C1-2, n=1)*  T spine (n=1)  L spine (n=1) |

HIV, human immunodeficiency virus; AIDS, acquired immune deficiency syndrome; POT, post-organ transplantation; PID, primary immunodeficiency
